# Derivation and validation of a clinical prediction rule for sleep apnoea syndrome for use in primary care

**DOI:** 10.3399/bjgpopen18X101481

**Published:** 2018-05-16

**Authors:** Erika Miranda Serrano, Amanda Lopez-Picado, Aitziber Etxagibel, Alfonso Casi, Laura Cancelo, Jose Ignacio Aguirregomoscorta, Itziar Menéndez, Monica Gonzalez, Felipe Aizpuru

**Affiliations:** 1 Research Fellow, Araba Research Unit, Araba University Hospital, OSI-Araba, Vitoria-Gasteiz, Spain; 2 Research Fellow, Araba Research Unit, Araba University Hospital, OSI-Araba, Vitoria-Gasteiz, Spain; 3 GP Graduate, Central Services, Vitoria-Gasteiz, Spain; 4 GP Graduate, Lakuabizkarra Health Centre, OSI-Araba, Vitoria-Gasteiz, Spain; 5 GP Graduate, Sleep Unit, Araba University Hospital, OSI-Araba, Vitoria-Gasteiz, Spain; 6 GP Graduate, Sleep Unit, Galdakao-Usansolo Hospital, Galdakao, Spain; 7 GP Graduate, Llodio Health Centre, OSI Barrualdea-Galdakao, Laudio Galdakao, Spain; 8 Head of Sleep Unit, Sleep Unit, Marques de Valdecilla University Hospital, Santander, Spain; 9 Research Fellow, Araba Research Unit, Araba University Hospital, OSI-Araba, Vitoria-Gasteiz, Spain

**Keywords:** sleep apnea, clinical predictive rule, primary health care

## Abstract

**Background:**

Several clinical prediction rules (CPRs) are available for sleep apnoea-hypopnoea syndrome (OSAH), but they are difficult to apply in primary care (PC).

**Aim:**

Derivation and validation of a CPR using simple measurements available in PC.

**Design & setting:**

A prospective study conducted in health centres from the area of influence of three Spanish hospitals.

**Method:**

Patients (aged 18–70 years) who attended for any reason; who presented with at least one of the three key symptoms for OSAH (snoring, breathing pauses while sleeping, and daytime sleepiness); and who were not undergoing non-invasive ventilation or prior treatment with continuous positive airway pressure (CPAP) were included. Anthropometric data, smoking habit, comorbidities, and Epworth test were collected. Patients were subsequently referred to the sleep unit (SU), where the decision was taken whether or not to instigate treatment. A multivariate logistic model was constructed using a sub-sample and scores assigned based on the regression coefficients; the CPR was validated with the remaining sample. Both receiver operating characteristic (ROC) curves were plotted and the sensitivity, specificity, and predictive values calculated.

**Results:**

The derivation sample comprised 352 patients, with 260 in the validation sample. The final factors (arterial hypertension [AHT], age, body mass index [BMI], and sex) were used to develop a rule with scores ranging from 0.00–5.50. The cut-off point that optimises the area under the curve (AUC) is ≥2.50 points (AUC = 0.78; sensitivity = 86%; specificity = 54%; positive predictive value [PPV] = 45%; negative predictive value [NPV] = 90%; likelihood ratio [LR] = 0.26). The properties for the validation sample with this cut-off point are as follows: AUC = 0.68; sensitivity = 81%; specificity = 43%; PPV = 61%; NPV = 68%; LR = 0.44.

**Conclusion:**

As in similar cases, the specificity is low, meaning that healthy people are referred to a specialist. A negative result rules out the disease in most cases.

## How this fits in

OSAH is associated with numerous diseases but only 5–9% of the population with severe OSAH is receiving treatment. Although various CPRs have been proposed, none of them is widely used. This CPR presents a lower sensitivity and specificity than other rules, and is more accessible and easier to use, making this rule an ideal tool for use in PC

## Introduction

OSAH affects 2–4% of the population.^1^ Its prevalence increases with age.^2^ Several studies have found an association between OSAH and numerous diseases, including hypertension,^1,3,4^ and cardiovascular^5^ and cerebrovascular disease.^6^ OSAH is associated with a worse quality of life,^7^ and a higher number of work^8^ and traffic accidents.^9,10^

Despite the risks associated with OSAH, only 5–9% of the population with severe OSAH is receiving treatment.^11^ Three key symptoms (snoring, pauses, and daytime sleepiness) have been used in standard clinical practice (SCP) to determine the probable presence of OSAH. However, none of these predicts the disease per se, as they are all very common in the general population.

This situation has outlined the need to develop a tool that discriminates patients presenting a more severe OSAH from those in whom the disease is not present or is unlikely to lead to its complications. Therefore, a tool like a CPR would be most useful for PC physicians, who could apply it to decide whether or not to refer a patient to specialised care.

A CPR is considered to be any decision-making instrument prepared from a minimum of three variables obtained from the case history, physical examination, or simple diagnostic tests.^12^ Although several attempts have been made to create a CPR,^13–16^ none of them is used in SCP. In some cases, this is because they use apnoea–hypopnoea index (AHI) values that are of little clinical interest (AHI = 5 or AHI = 10)^17^ or, in the case of the neck circumference, due to the collinearity with BMI, which is in the final model as current guidelines indicate the use of CPAP for AHI values >30, or 15 if accompanied by related comorbidity or severe symptomatology.

Kushida *et al* ^15^reported a high-quality prediction (sensitivity = 98%; specificity = 100%), but this rule is difficult to apply in SCP as the collection of the variables requires meticulous training. Chai-Coetzer *et al* ^17^ developed the OSA50 screening tool plus overnight oximetry, which shows a very good NPV, with the drawback for its use in PC being that it requires an at-home overnight test.

This study proposes the derivation and validation of a prediction rule, using measurements easy to obtain in PC, in order to distinguish between patients at high and low risk of suffering OSAH.

## Method

### Design and patients

The CPR was developed using a sample of patients recruited prospectively between February 2011 and August 2012. The derivation sample comprised patients who attended the PC departments of two urban health centres and were referred to the SU at the Araba University Hospital. Patients of the validation sample were recruited at various health centres and referred to the Hospital de Galdakao-Usansolo and the University Hospital in Valdecilla (Santander).

All subjects aged 18–70 years and presenting at least one of the three key symptoms for OSAH (snoring, daytime sleepiness, or breathing pauses while asleep) were recruited on spontaneous consultation of a PC physician for any reason. Patients with a previous diagnosis of OSAH, prior CPAP treatment or non-invasive mechanic ventilation were excluded. Participants provided consent to participate in the study.

### Data collection

After collection of the information in the PC centres, participants were asked to attend the SU at the corresponding hospital. The doctor at the SU, blinded to the information collected in the PC setting, made the diagnostic decisions (polysomnography [PSG], respiratory polygraphy [RP], both, or neither) as well as the therapeutic decisions (CPAP, mandibular advancement device [MAD], postural device, or hygiene or dietary measures), following SCP.

### Reliability analysis

The only information obtained in both the PC centre and the SU, the BMI, was used for the inter-observer reliability analysis in order to validate the information collected by the PC physicians.

### Variables

The characteristic to be predicted is the clinical decision of the sleep specialist as regards indication for specific diagnostic tests (PSG, RP, or both tests); and indication of treatment (CPAP, MAD, or a postural device).

The predictive variables taken into consideration were sex, age, weight, height, neck circumference, snoring (five or more times per week), breathing pauses while asleep reported by the partner or companion, daytime hypersomnia, accidents in the past year due to drowsiness, morning tiredness, morning sensation of asphyxia, AHT, heart failure, diabetes mellitus (DM), smoking habit, alcohol consumption, and daytime sleepiness according to the Epworth scale.

In addition, BMI, neck circumference, and Epworth scale were collected at the SUs. Along with this information, global AHI, supine AHI, and T90 (the percentage of time during which arterial O_2_ saturation is <90%) were obtained if available from the PSG and/or RP reports.

### Sample size

According to Flahault *et al,* ^18^ approximately 298 cases with the disease, or 750 subjects, are required to estimate an expected sensitivity for the test of 0.95 with a confidence limit (95%) of not less than 0.90, considering a prevalence of 40% for the disease in the study population (data from the SU in Vitoria).

Two hundred subjects are required for the validation phase for a power of 90% to confirm the hypothesis that the sensitivity of the CPR in the validation population does not differ from that obtained for the derivation sample by >3%.

### Statistical analysis

#### Treatment of variables

Means and standard deviations were used to describe continuous variables, and frequencies and percentages for categorical variables.

Continuous variables were categorised in order to be applied in a simple manner. Looking at the distribution of results, values close to the median or values close to the tertiles were used as cut-off points.

Characteristics of the patients in the derivation and validation samples were compared using the X^2^ test or the student’s *t*-test for continuous variables.

SPSS (version 22) and R freeware (version 3.1.1) were used for all analyses.

### Derivation of the prediction rule

According to Kharbanda *et al,* ^19^ those variables with <10% lost values were selected as potential predictive variables, which did not exclude any of those from the model. The characteristics of treated patients were compared with those of untreated patients using a univariate logistic regression. A value of *P*<0.2 indicated that a variable was potentially predictive and should be taken into consideration during the multivariate analysis.

Subsequently, the final multivariate logistic regression model was described using a stepwise variable selection method and the LR test to compare two models. The criterion for introducing variables into the model was *P*≤0.05.

A backward stepwise model was also prepared using the LR test in order to ensure the most parsimonious rule possible. The criterion for excluding variables from the model was *P*>0.05. Both variable selection criteria led to the same final model. The goodness-of-fit was evaluated using the Hosmer–Lemeshow test.

The final scores for the prediction rule were obtained from the logistic regression coefficients using the lowest risk category as reference.^19^

### Predictive ability and validation of the rule

The predictive ability of the rule was evaluated for each sample using ROC curves, which present the decision reached with respect to the score. To evaluate the predictive accuracy of the rule for the derivation and validation samples, different cut-off points were established and 2×2 tables were constructed to calculate the following measures: sensitivity, specificity, PPVs, and NPVs (including 95% confidence intervals [CI]). Table 1 shows the CPR in the format it would be used in PC.

**Table 1.** Clinical prediction rule in the format it would be used in primary care

		Points
**Age, years**	18–45	0.00
	46–59	1.25
	60–70	1.50
**BMI**	<30	0.00
	≥30	1.50
**Sex**	Female	0.00
	Male	1.50

BMI = body mass index.

### Reliability analysis

The kappa statistic was calculated to compare the interobserver reliability for measurement of each of the variables included in the CPR. A kappa value >0.60 was considered to be acceptable.

## Results

**Figure 1.**
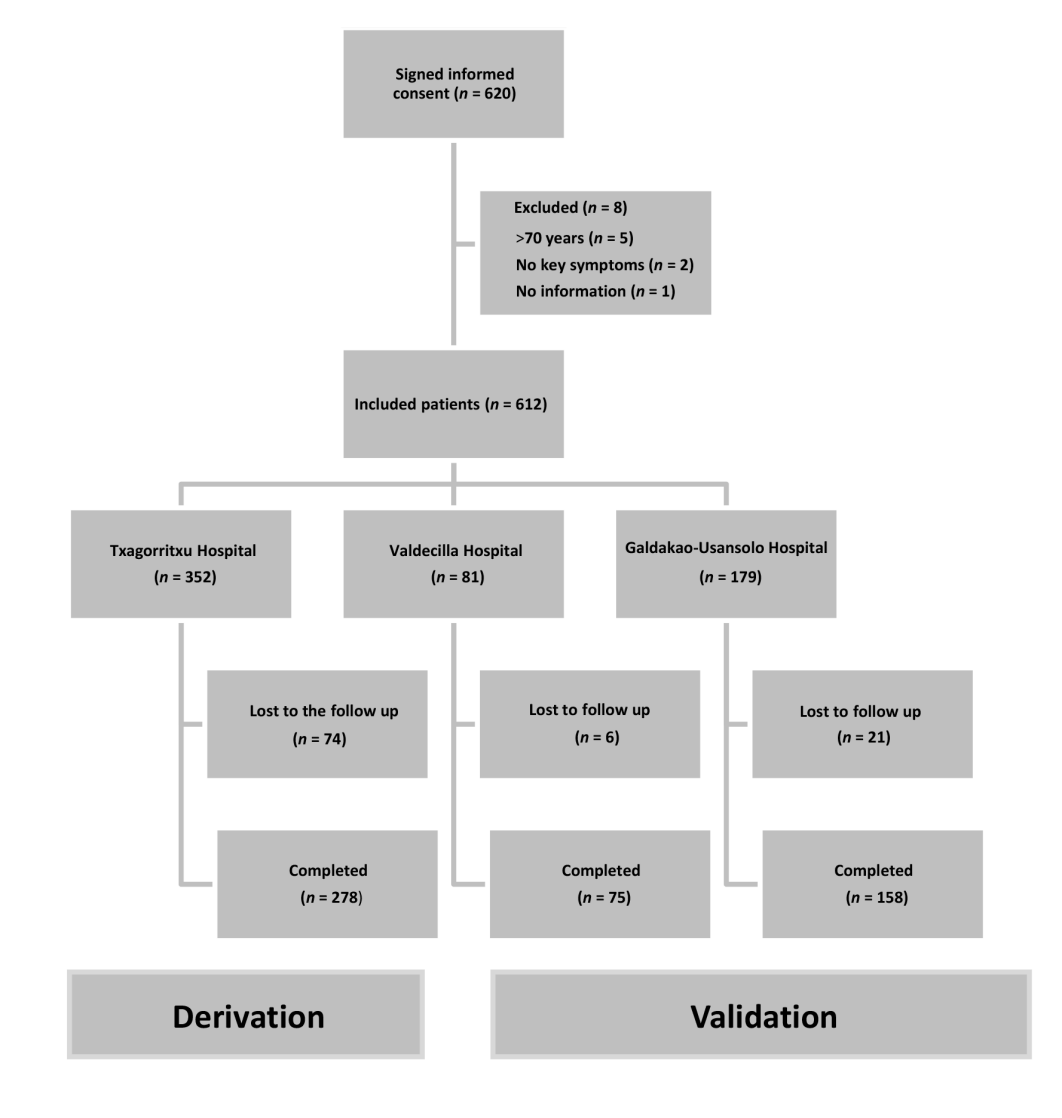
Flow chart of inclusion and exclusion criteria

Informed consent was obtained from 620 patients. Eight were excluded: five because they were aged >75 years; two because they did not have any of the key symptoms; and one for technical reasons. Full information (predictive variables, and indication for diagnostic tests and/or treatment) was available for 278 of the 352 subjects in the derivation sample (79.0%) and 233 of the 260 subjects (89.6%) in the validation sample (Figure 1). The complementary examination was conducted for 226 of the 231 subjects from the validation sample who attended the SU (97.8%), and 122 of them (54.0%) had an indication for treatment with CPAP or another device (*P*<0.001).

Table 2 shows the characteristics of both samples. Differences can be seen between validation and derivation samples regarding sex, BMI, breathing pauses, daytime hypersomnia, history of accidents due to drowsiness, morning tiredness, sensation of asphyxia, neck circumference, AHT, DM, smoking habit, score on the Epworth scale, and final decision by the SU specialist.

**Table 2.** Comparison of the derivation and validation samples

	Derivation (*n* = 352), *n* (%)	Validation (*n* = 260), *n* (%)	*P* value
Primary care data complete	350 (99.4)	249 (95.8)	0.002
Lack of sleep	74 (21.0)	27 (10.4)	0.001
Male	122 (34.7)	67 (25.8)	0.019
Mean age, SD	48.84 (11.15)	49.01 (11.55)	0.852
Mean weight, SD	81.11 (16.20)	86.66 (17.34)	<0.001
Mean height, SD	169.45 (9.70)	169.03 (8.96)	0.588
Mean BMI, SD	28.18 (4.88)	30.26 (5.24)	<0.001
Frequent snoring	346 (98.3)	257 (98.8)	0.740
Breathing pauses	134 (38.1)	182 (70.0)	<0.001
Daytime hypersomnia	149 (42.3)	163 (62.7)	<0.001
History of accidents	12 (3.4)	22 (8.5)	0.007
Morning tiredness	151 (42.9)	157 (60.4)	<0.001
Waking with sensation of asphyxia	68 (19.3)	96 (36.9)	<0.001
Mean neck circumference, SD	39.08 (4.27)	40.09 (4.09)	0.003
AHT	79 (22.4)	89 (34.2)	0.001
Heart failure	3 (0.9)	7 (2.7)	0.106
DM	20 (5.7)	33 (12.7)	0.002
**Smoking habit**			
Non-smoker	131 (37.2%)	74 (28.5%)	0.027
Smoker	106 (30.1%)	76 (29.2%)	
Ex-smoker	115 (32.7%)	110 (42.3%)	
Drinks alcohol	126 (35.8%)	111 (42.7%)	0.083
Mean Epworth score, SD	9.24 (4.14)	10.04 (4.90)	0.034
**Diagnostic test**			
CPSG	11 (3.8)	159 (68.8)	<0.001
RP	272 (92.8)	67 (29.0)	
None	6 (2.0)	5 (2.2)	
Both RP and CPSG	4 (1.4)	0 (0.0)	
**Therapeutic decision**			
CPAP	79 (28.5)	118 (50.6)	<0.001
No CPAP	192 (69.3)	111 (47.6)	
Mandibular advancement device	4 (1.4)	4 (1.7)	
Postural device	2 (0.7)	0 (0.0)	

AHT = arterial hypertension. BMI = body mass index. CPAP = continuous positive airway pressure. CPSG = conventional polysomnography. DM = diabetes mellitus. RP = respiratory polygraphy. SD = standard deviation.

Table 3 shows the result of the univariate analysis, in which the risk factors were compared with the therapeutic decision. Subjects requiring treatment were more likely to be older, male, obese, with larger neck circumferences, and with chronic disease.

**Table 3.** Derivation sample. Univariate logistic regression for the primary variable 'therapeutic decision'. Selection of variables for the final model.

Variable	OR	95% CI	*P* value
Male	3.15	1.74 to 5.99	<0.001
Age 18–45; 46–59; 60–70 (1)	2.56	1.37 to 4.95	0.002
Age 18–45; 46–59; 60–70 (2)	3.15	1.52 to 6.68	
BMI <30; ≥30	3.61	2.11 to 6.24	<0.001
Frequent snoring	0.66	0.11 to 5.07	0.656
Breathing pauses	1.92	1.14 to 3.23	0.013
Daytime hypersomnia	1.06	0.63 to 1.77	0.835
History of accidents	0.31	0.02 to 1.81	0.220
Morning tiredness	0.62	0.36 to 1.04	0.071
Waking with sensation of asphyxia	1.02	0.54 to 1.89	0.948
Neck circumference ≤38; 38.1–42; >42 (1)	2.04	1.07 to 3.99	<0.001
Neck circumference ≤38; 38.1–42; >42 (2)	7.20	3.57 to 15.08	
AHT	4.00	2.26 to 7.17	<0.001
DM	3.36	1.04 to 11.64	0.043
Smoking status (1)	1.08	0.56 to 2.09	0.122
Smoking status (2)	1.80	0.99 to 3.32	
Alcohol status	1.07	0.62 to 1.81	0.816
Epworth score <9; ≥9	1.67	0.99 to 2.86	0.056

AHT = arterial hypertension. BMI = body mass index. CI = confidence intervals. DM = diabetes mellitus. OR = odds ratio.

The final multivariate model (Hosmer–Lemeshow goodness-of-fit *P* = 0.903), can be seen in Table 4, with the variables included and the coefficients estimated, along with the scores for the CPR calculated using these coefficients. According to this rule, 1.50 points were awarded each to males, to obese patients, and to those aged >60 years. Subjects aged 46–59 years were awarded 1.25 points, with an additional point for those who were hypertensive. Therefore, the total score for the rule ranges between 0.00 and 5.50 points. 

**Table 4.** Derivation sample. Final logistic regression and scores for the clinical prediction rule.

	B	SE	Significance	Exp(B)	95% CI for Exp(B)	β_i/_ β_min_	Score
AHT	0.9150	0.3304	0.00562	2.497	1.306 to 4.787	1	1.00
Age 46–59	1.1955	0.3746	0.00141	3.305	1.614 to 7.055	1.30657	1.25
Age 60–70	1.4440	0.4421	0.00109	4.238	1.804 to 10.281	1.57814	1.50
BMI ≥30	1.4490	0.3192	5.65e–06	4.259	2.300 to 8.074	1.58360	1.50
Male	1.3740	0.3488	8.18e–05	3.951	2.040 to 8.059	1.50164	1.50
Constant	−2.0823	0.3435	1.34e–09	0.125	0.061 to 0.236		

Hosmer–Lemeshow test *P* = 0.903.

AHT = arterial hypertension. BMI = body mass index. CI = confidence interval. SE = standard error.

**Figure 2.**
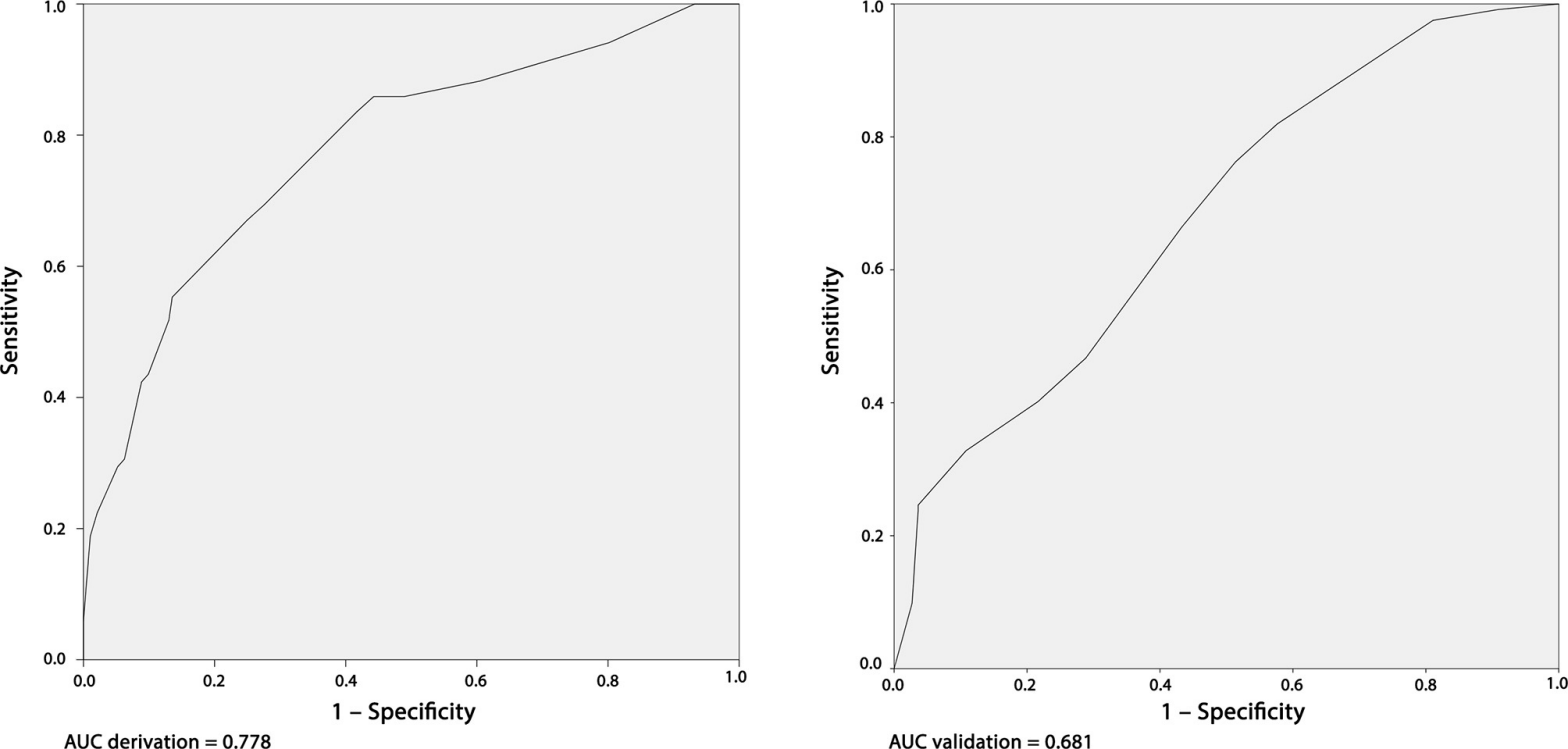
Receiver operating characteristic curves for the derivation and validation samples. AUC = area under curve.

The AUCs for the derivation sample (77.8%) and for the validation sample (68.1%) can be seen plotted in Figure 2.

The sensitivity, specificity, PPV, and NPV for the different cut-off points on the scale are provided in Table 5. To guarantee a high sensitivity, and therefore the detection of patients who require treatment, cut-off points of 1.50, 2.25, 2.50, or 2.75 are proposed as possible discriminatory values. The rule has a better diagnostic ability for the derivation than for the validation sample. However, selection of the most conservative cut-off point possible (1.50) leads to a sensitivity of 97.5% for the derivation sample, although this implies that only 24 of 233 (10.3%) subjects attending a PC are free of diagnosis.

**Table 5.** Different cut-off points for the clinical prediction rule.

	Cut-off point	Treated (*N* = 85)	Not treated (*N* = 192)	Sensitivity, % (95% CI)	Specificity, % (95% CI)	PPV, % (95% CI)	NPV, % (95% CI)
Derivation (*n* = 277)	≥1.50	80	154	94.1 (89.1 to 99.1)	19.8 (14.2 to 25.4)	34.2 (28.1 to 40.3)	88.4 (78.8 to 98.0)
	≥2.25	73	94	85.9 (78.5 to 93.3)	51.0 (44.0 to 58.1)	43.7 (36.2 to 51.2)	89.1 (83.3 to 94.9)
	≥2.50	73	88	85.9 (78.5to 93.3)	54.2 (47.1 to 61.2)	45.3 (37.7 to 53.0)	89.7 (84.1 to 95.2)
	≥2.75	71	80	83.5 (75.6 to 91.4)	58.3 (51.4 to 65.3)	47.0 (39.1 to 55.0)	88.9 (83.4 to 94.4)
	**Cut-off point**	**Treated** **(*N* = 122)**	**Not treated** **(*N* = 111)**	**Sensitivity, %** **(95% CI)**	**Specificity, %** **(95% CI)**	**PPV, %** **(95% CI)**	**NPV, %** **(95% CI)**
Validation (*n* = 233)	≥1.50	119	90	97.5 (94.8 to 100.3)	18.9 (11.6 to 26.2)	56.9 (50.2 to 63.7)	87.5 (74.3 to 100.7)
	≥2.25	100	64	82.0 (75.1 to 88.8)	42.3 (33.2 to 51.5)	61.0 (53.5 to 68.4)	68.1 (57.1 to 79.1)
	≥2.50	99	63	81.1 (74.2 to 88.1)	43.2 (34.0 to 52.5)	61.1 (53.6 to 68.6)	67.6 (56.7 to 78.5)
	≥2.75	93	57	76.2 (68.7 to 83.8)	48.6 (39.4 to 57.9)	62.0 (54.2 to 69.8)	65.1 (54.8 to 75.3)

For the reliability, the kappa index for this measure is 0.834, although it should be noted that this analysis was performed for a total of 334 out of 612 cases (54.6% of the total sample).

## Discussion

### Summary

This study has derived and validated a CPR for identifying patients with suspected OSAH in PC centres. Four independent predictive factors (AHT, age >46 years, BMI ≥30, and male sex) were selected, and a rule ranging from 0.00 to 5.50 points was generated combining them. Despite being variables 'classically' associated with the presence of OSAH, neck circumference and breathing pauses do not form part of the final model as these variables are confounded by sex and, in the case of the neck circumference, due to the collinearity with BMI, which is in the final model.

Although the final goal of a prediction rule is to obtain the highest possible sensitivity, in this study two cut-off points are proposed for two different scenarios. In this case, selecting a cut-off point of ≥1.50 points (highest sensitivity for the rule with the derivation sample) means that sleep tests must be performed in a large number of healthy patients due to the low specificity. This implies the overuse of already limited resources, in addition to the indirect costs and inconveniences generated to the patient.

The cut-off point that achieves the best results in terms of sensitivity and specificity for the derivation sample is ≥2.50 points, with a lower sensitivity counterbalanced by a marked increase in specificity.

### Strengths and limitations

However, selection of this cut-off point for the validation sample leads to markedly different results as both sensitivity and specificity decrease. This nevertheless appears to be the most reasonable option in light of the results. The PPV of the validation sample is also 15–20% higher.

This is likely to be mainly due to the fact that the derivation and validation populations sampled differ in terms of baseline risk profile regarding the disease. Thus, the derivation sample includes a higher proportion of males, with a higher BMI and, in general, a higher number of key symptoms for the disease, and therefore a higher pre-test probability.

The main limitation of this study is the difference between the derivation and validation groups; the values obtained during validation are markedly lower than those obtained during derivation of this rule. However, this situation merely reflects the study's setting, where access to some healthcare services is unequal and depends on the healthcare organisation.

The present authors also decided to limit the study to population those aged <70 years, with the aim of identifying the disease in a population with a low prior probability of having it. It is doubtful that a rule valid for asymptomatic people works the same way in an aged population, with a higher probability and a greater possibility of confounding due to comorbidity. Similar studies have also chosen this limit, notably the aforementioned work by Chai-Coetzer.^17^

### Comparison with existing literature

Although the results obtained do not demonstrate as high a sensitivity and specificity as the prediction rules developed previously,^14–17^ the present authors believe that the studied rule is easier to apply in PC settings, resulting in a more rational referral to specialised units. This means that the rule is useful despite the drawbacks discussed above.

A recent study conducted in the UK^20^ surveyed PC physicians regarding the use of prediction rules in clinical practice. The results showed that a high percentage of physicians were unaware of and/or did not use prediction rules widely validated in PC, as they considered these rules to be of little use, or preferred their own medical judgement. This could possibly be explained by the complexity of such rules. Therefore, a rule that is easy to use could easily be implemented in already saturated PC centers.

### Implications for practice

In conclusion, this study presents a CPR for diagnosing OSAH which, despite presenting a lower sensitivity and specificity than other such rules, is more accessible and easier to use. This makes it an ideal tool for use in PC, allowing the referral of patients susceptible to presenting the disease to SUs while ensuring a more rational use of the resources available.
